# Supplementary material for: Presence of Mercury in an Arid Zone of Mexico: A Perspective Based on Biomonitoring of Mammals from Three Trophic Guilds
Source: Biology (Basel). 2024 Oct 11;13(10):811. doi: 10.3390/biology13100811 (PMC11504424; doi:10.3390/biology13100811)
Supplement: Supplementary file 1 [file biology-13-00811-s001.zip › Supplementary material. Appendix S1.pdf]

Appendix S1. Mercury content in three mammalian species from Xoxafí, Mexico (Nt=49).

Hg content (ppb)	Matrix	Species	Season
1. 4.944	Liver	*C. townsendii*	Dry
2. 5.064	Liver	*C. townsendii*	Dry
3. 7.925	Liver	*C. townsendii*	Dry
4. 7.598	Liver	*C. townsendii*	Dry
5. 8.173	Hair	*C. townsendii*	Dry
6. 8.252	Hair	*C. townsendii*	Dry
7. 8.803	Hair	*C. townsendii*	Dry
8. 7.875	Hair	*C. townsendii*	Dry
9. 1.719	Liver	*C. townsendii*	Rainy
10. 1.938	Liver	*C. townsendii*	Rainy
11. 1.375	Liver	*C. townsendii*	Rainy
12. 2.125	Liver	*C. townsendii*	Rainy
13. 1.625	Liver	*C. townsendii*	Rainy
14. 11.688	Hair	*C. townsendii*	Rainy
15. 12.438	Hair	*C. townsendii*	Rainy
16. 12.188	Hair	*C. townsendii*	Rainy
17. 10.375	Hair	*C. townsendii*	Rainy
18. 1.188	Liver	*P. melanophrys*	Rainy
19. 1.406	Liver	*P. melanophrys*	Rainy
20. 4.219	Liver	*P. melanophrys*	Rainy
21. 1.625	Liver	*P. melanophrys*	Rainy
22. 1.438	Liver	*P. melanophrys*	Rainy
23. 7.197	Liver	*P. melanophrys*	Dry
24. 6.719	Liver	*P. melanophrys*	Dry
25. 4.353	Liver	*P. melanophrys*	Dry
26. 7.133	Liver	*P. melanophrys*	Dry
27. 8.125	Liver	*P. melanophrys*	Dry
28. 7.666	Liver	*P. melanophrys*	Dry
29. 10.941	Hair	*P. melanophrys*	Rainy
30. 6.269	Hair	*P. melanophrys*	Rainy
31. 5.069	Hair	*P. melanophrys*	Rainy
32. 5.033	Hair	*P. melanophrys*	Rainy
33. 6.912	Hair	*P. melanophrys*	Rainy
34. 7.197	Hair	*P. melanophrys*	Dry
35. 6.719	Hair	*P. melanophrys*	Dry
36. 4.353	Hair	*P. melanophrys*	Dry
37. 7.133	Hair	*P. melanophrys*	Dry
38. 8.125	Hair	*P. melanophrys*	Dry
39. 7.666	Hair	*P. melanophrys*	Dry
40. 7.567	Liver	*L. yerbabuenae*	Dry

41.	4.755	Liver	*L. yerbabuenae*	Dry
42.	4.266	Liver	*L. yerbabuenae*	Dry
43.	6.038	Liver	*L. yerbabuenae*	Dry
44.	6.491	Liver	*L. yerbabuenae*	Dry
45.	8.928	Hair	*L. yerbabuenae*	Dry
46.	8.956	Hair	*L. yerbabuenae*	Dry
47.	9.995	Hair	*L. yerbabuenae*	Dry
48.	8.264	Hair	*L. yerbabuenae*	Dry
49.	9.639	Hair	*L. yerbabuenae*	Dry
