# Supplementary material for: Presence of Mercury in an Arid Zone of Mexico: A Perspective Based on Biomonitoring of Mammals from Three Trophic Guilds
Source: Biology (Basel). 2024 Oct 11;13(10):811. doi: 10.3390/biology13100811 (PMC11504424; doi:10.3390/biology13100811)
Supplement: Supplementary file 1 [file biology-13-00811-s001.zip › Supplementary material. Appendix S2.pdf]

Appendix S2. Method validation (Nt=49).

Coeficcient of correlation (r^2)	0.999
Coefficient of variation	0.907
Mean absorbance of calibration curves	0.042
Standard deviation of calibration curves	0.038
Limit of detection	0.158 ppb
Limit of quantification	0.428 ppb
Uncertainty	0.005
Reproducibility	(4.21 ± 1.29) %
Repetibility	(0.2 ± 0.005) %
Sensibility	(0.009 ± 0.0002) ppb
Lineal interval	0.5-10 ppm
